# Effects of Parity and Postpartum Depression on Mother-Infant Bonding in the First Month Postpartum: A Retrospective Study

**DOI:** 10.7759/cureus.45585

**Published:** 2023-09-20

**Authors:** Keita Kawai, Hiroi Tomioka, Hiroki Yamada, Sho Mamiya, Azumi Kato, Akira Iwanami, Atsuko Inamoto

**Affiliations:** 1 Mental Care Center, Showa University Northern Yokohama Hospital, Kanagawa, JPN; 2 Department of Psychiatry, Showa University, Tokyo, JPN; 3 Department of Obstetrics and Gynecology, Showa University Northern Yokohama Hospital, Kanagawa, JPN

**Keywords:** abuse prevention, perinatal mental health, postpartum depression, parity, mother–infant bonding

## Abstract

Objective

This study aimed to examine the relationship between parity, postpartum depression (PPD), and mother-infant bonding (MIB) failure in the first month postpartum.

Methods

The study included 1,509 Japanese patients (748 primiparous and 761 multiparous). MIB was assessed using the Mother-to-Infant Bonding Scale Japanese version (MIBS-J), which was translated in 2012, and its subscales, including lack of affection (LA) and anger and rejection (AR). Postpartum depression (PPD) was assessed using the Japanese version of the Edinburgh Postnatal Depression Scale (EPDS) and its subscales, including anxiety (ANX), anhedonia (ANH), and depression (DEP). Multiple regression analyses using interaction terms were performed to examine the association of parity with the MIBS-J and EPDS.

Results

Parity was significantly associated with AR. ANX and ANH were strongly associated with LA, and ANX and DEP were strongly associated with AR. The interaction term “parity×EPDS total” was significantly associated with MIBS-J total, LA, and AR scores.

Conclusions

Primiparas and mothers with high ANX had more negative emotions toward their children during the first month postpartum, and mothers with high ANX or ANH had less interest in their children.

## Introduction

Mother-infant bonding (MIB) is the emotional bond between a mother and her infant. MIB failure can intensify a mother's negative feelings towards her child and increase the risk of aggressive impulses and inappropriate childcare [1,2]. Brockington IF et al. [3] reported that while postpartum depression (PPD) has previously been used as a predictor of abuse and inappropriate childcare behavior, MIB failure might be a more appropriate indicator. The Mother-to-Infant Bonding Scale Japanese version (MIBS-J) and two of its subscales, namely, lack of affection (LA) and anger and rejection (AR) [4], are widely used in Japan to measure MIB. The Mother-to-Infant Bonding Questionnaire (MIBQ) [5] developed by Kumar RC [2] included a "scared or panicky" item, and Yoshida K et al. [4] translated it into Japanese as the MIBS-J. In addition, Taylor A et al. [6] removed the item "possessive" from the MIBQ and created the Mother-to-Infant Bonding Scale (MIBS) as another measurement tool. The MIBS response options gauge the intensity of emotions, whereas the MIBS-J response options assess both the intensity and the frequency of feelings. In Japan, primipara is a risk factor for MIB failure [7-10]. A detailed study that used subscales showed that during the first year postpartum, being primipara was associated with a higher MIBS-J total score and AR score but not with LA score [10]. In addition, in the first month postpartum, being primipara was associated with higher MIBS-J total scores [7,8]. Tsuchida A et al. [9] assessed the differences between the birth experiences of mothers who had given birth twice. They reported significant decreases in both ANX about caregiving and lack of maternal feeling (Table 1) at the first month postpartum. Fukui N et al. [7] reported that being primipara increases both LA and AR scores, i.e., scores of subscales developed by Motegi T et al. [11], in the first month of postpartum. In Japan, primipara is also a risk factor for PPD [7-10,12,13]. Previous studies have shown that primiparas with PPD strongly felt a lack of support from friends [14], and mothers who experienced depression in early pregnancy showed that the presence of supportive people (in terms of number of supporters) a mother has during pregnancy strongly helped in reducing depressive symptoms during the first month postpartum [15]. These results indicate a modification effect between parity, depression, and social support, indicating the possibility that MIB failure, closely related to PPD [16], has a modification effect on these factors.

At different postpartum intervals, PPD and MIB have been found to be related [6,9,17]. Yoshida K et al. [4] reported that PPD correlated with both LA and AR scores at five days, one month, and four months postpartum. Further, Fukui N et al. [7] reported that in the first month postpartum, depression predicted both LA and AR scores, but anxiety predicted only AR scores. Ohara M et al. [18] reported that PPD symptoms correlated with the LA and AR scores of the MIBQ at the first month postpartum. Kasamatsu H et al. [19] reported that PPD symptoms at one and six months postpartum were associated with LA and AR scores in the first year postpartum. By examining the relationship between MIB, PPD, and parity using the widely used MIBS-J subscale developed by Yoshida K et al. [4], we can potentially enhance the generalizability of our findings and deepen our comprehension of MIB. The better we grasp the modifying effects of MIB, the more precise our understanding becomes of the specific maternal characteristics warranting special attention. A collaborative study conducted across 163 institutions in Japan found that out of 118 children whose deaths between 2014 and 2016 were considered likely due to child abuse, 74 (62.7%) were less than a year old [20]. This underscores the urgent need for abuse prevention starting from the early postpartum period. However, while MIB failure is associated with abuse, it is not mentioned in the Diagnostic and Statistical Manual of Mental Disorders, 5th Edition (DSM-5) or the International Classification of Diseases 10th Revision (ICD-10). This concept's significance has recently gained attention, resulting in relatively limited research despite a recent surge [17]. This study aimed to explore the relationship between parity, PPD, and MIB failure during the first month postpartum. We centered our investigation on the relationship between parity and PPD in this initial postpartum month, given it's the peak onset time for PPD in the Japanese population [12]. This period also aligns with routine medical checkups. Our goal was to gain a deeper understanding of MIB failure in these early stages after childbirth. By doing so, we hope to facilitate the development of strategies that offer appropriate support for mothers struggling with MIB failure and various emotional challenges, thereby preventing potential abuse and enhancing maternal mental healthcare.

## Materials and methods

Research data

This study was conducted at Showa University Northern Yokohama Hospital, a large general hospital with an emergency psychiatric inpatient ward in urban Japan. This retrospective cohort study was designed in accordance with the Declaration of Helsinki and approved by the Clinical Trial Review Committee of Showa University Northern Yokohama Hospital (approval number: 20H042). Medical data were collected retrospectively from electronic medical records. Instead of being exempted from the requirement for informed consent, we only included patients' data after they were given the opportunity to refuse consent by opting out. To safeguard patient confidentiality, all participant data have been de-identified. We have considered the Strengthening the Reporting of Observational Studies in Epidemiology (STROBE) guidelines to the best of our ability for reporting this retrospective study [21].

Participants

The study participants were Japanese women who delivered at Showa University Northern Yokohama Hospital between January 2020 and September 2021. Only the first delivery was included if a woman gave birth more than once during this period. Of the 1,816 women included in the study, cases of 52 foreigners, 46 stillbirths, 69 multiple pregnancies, and 29 births with babies weighing <1,500 g, as well as 22 women that could not be followed up or were not available due to transfer to another hospital after delivery and 89 cases with missing measurement data were excluded. Finally, the data of 1,509 women were included for analysis.

Measures

Japanese Version of the Mother-to-Infant Bonding Scale

The primary endpoint of this study was the severity of MIB failure in the first month postpartum. The MIBS-J is a 10-item self-administered questionnaire scored on a 4-point Likert scale ranging from 0 to 3. Good validity and reliability have been reported for the MIBS-J [4]. Typically, higher scores indicate greater severity of MIB failure. The MIBS-J total scores can range from 0 to 30, while both the LA and AR scores can vary from 0 to 12. In this study, the MIBS-J total score, as well as the LA and AR scores (Table 1), were treated as continuous variables and used as dependent variables. The MIBS-J evaluates bonding feelings at the time of measurement (around one month postpartum), setting it apart from Taylor's MIBS [6], which asks mothers to recall their feelings "in the first few weeks" postpartum. The various subscales of the MIBS-J and MIBQ are detailed in Table 1.

**Table 1:** The subscales of the MIBS-J and MIBQ. MIBS-J: Mother-to-Infant Bonding Scale Japanese Version; MIBQ: Mother-to-Infant Bonding Questionnaire; LA: Lack of Affection; AR: Anger and Rejection; LMF Lack of Maternal Feeling; AC: Anxiety about Caregiving. Emotions are indicated by the numbers. MIBQ: 1. Loving, 3. Neutral and felt nothing, 4. Possessive, 5.Resentful, 6. Dislike, 7. Protective, 8. Joyful. MIBS-J: 1. Loving, 2. Scared or panicky, 3.Resentful, 4. Neutral and felt nothing, 5.Aggressive, 6.Joyful, 7. Dislike, 8. Protective, 9. Disappointed, 10. Possessive.

Original Scale	MIBQ		MIBS-J					
Subscale study	Ohara M et al. [5]		Tsuchida A et al. [9]		Motegi T et al. [11]		Yoshida K et al. [4]	
Subscale name	LA	AR	LMF	AC	LA	AR	LA	AR
Measured emotions	1, 3, 4, 7, 8	5, 6	1, 4, 9	2, 6	1, 4, 6, 8, 10	3, 5, 9	1, 6, 8, 10	2, 3, 5, 7
Study, used by	Ohara M et al. [18]		Tsuchida A et al. [9]		Fukui N et al. [7]		The present study	

Japanese Version of the Edinburgh Postnatal Depression Scale

We used the scores of the Edinburgh Postnatal Depression Scale (EPDS) [22] translated into Japanese by Okano T et al. [23] at the one-month postpartum checkup to evaluate postpartum depression. The EPDS is a self-administered questionnaire comprising 10 items and is scored on a 4-point Likert scale from 0 to 3. Generally, a higher score indicates a higher severity of depression. Different cut-off points for diagnosing PPD are set in different countries, and a cut-off point of 8/9 is widely used in Japan [23]. In addition, the EPDS has been shown to have a three-factor structure of ANX, ANH, and DEP, with items 3, 4, and 5 for ANX; items 1 and 2 for ANH; and items 7, 9, and 10 for DEP [24,25]. The EPDS total scores range from 0 to 30, the ANX and DEP scores range from 0 to 9, and the ANH score ranges from 0 to 6. This study analyzed the EPDS total scores and the ANX, ANH, and DEP scores as continuous variables.

Social Support

In this study, the following five questions, adopted from a pool of 11 sets of questions, were asked during the one-month postpartum checkup. These questions were aimed at assessing the dimensions of childcare support and associated challenges in order to measure social support: "4-①: Can you confide in your husband about anything?", "4-②. Can you confide in your mother about anything?", "4-③. Besides your husband and mother, is there anyone else you can talk to?", "5. Do you have any difficulties in your life or financial worries?" and "6. Are you satisfied with your current residence and environment for raising children?". For social support, since the number of supporters and level of satisfaction with the support received have been shown to have different effects on PPD [15], we decided to create two items for social support in this study. For questions 4-①, 4-②, and 4-③, the total number of "yes" responses, representing the number of supporters for that person, was denoted as "number of supporters" and was treated as a continuous variable. Participants indicating support issues were those who responded "having a problem" to either Questions 5 or 6, and the responses were treated as categorical variables.

Covariates

Based on previous studies on MIB failure, we selected age, history of mental disorders, history of miscarriage, parity, type of delivery (vaginal, instrumental, planned cesarean section, and emergency cesarean section), premature birth (<37 weeks), the child's sex, and admission to a neonatal intensive care unit or growing care unit as covariates [10,26-29].

Statistical analysis

Descriptive Statistics and Univariate Analysis

Data for all variables in this study are presented according to parity groups as well as for all participants. Continuous variables are reported as mean and SD, while categorical variables are given as the number of persons and their respective proportions. Univariate analysis was also performed to compare parity groups. Because of the possible influence of measurement timing on MIB and PPD, we examined the correlation of the MIBS-J total and EPDS total scores with the number of postpartum days at the time the measurements were taken.

Multiple Regression Analysis 1

We examined the association between PPD symptoms, parity, social support, and MIB failure. The MIBS-J total, LA, and AR scores were set as dependent variables, and the analysis was adjusted for the abovementioned covariates without parity. Furthermore, multiple regression analysis was performed by adjusting for parity, EPDS total score, number of supporters, and problems with support in addition to the above covariates. The variance inflation factor (VIF) was also calculated to examine multicollinearity.

Effect Modification

We investigated the modifying effect of MIB failure on the interaction between parity, PPD, and social support. Initially, we formulated three interaction terms: "parity×EPDS Total," "parity×number of supporters," and "parity×problems with support." Subsequently, these three interaction terms were integrated into the three multiple regression models from multiple regression analysis 1 to assess effect modification. For interaction terms with significant differences, we graphed simple linear regression lines and visually represented the disparity in slopes based on parity. To further evaluate the variance in the slopes of these lines, we executed a multiple regression analysis using the dependent variables. This analysis considered three independent variables: the significant interaction term, parity, and the other variable included in that interaction term.

Multiple Regression Analysis 2

The relationship between the three subscales of the EPDS and MIBS-J, LA subscale, and AR subscale was examined. First, a multiple regression model was created using the MIBS-J total, LA, and AR scores as dependent variables and "ANX," "ANH," and "DEP" as independent variables (crude model). We further performed a multiple regression analysis, adjusting for "parity," "number of supporters," "problems with support," and all the abovementioned covariates (adjusted model).

The significance level for this study was set at p<0.05 with two-tailed tests. All statistical analyses were conducted using the SPSS version 25 (IBM Japan, Tokyo, Japan).

## Results

Descriptive statistics and univariate analysis

The descriptive statistics for the 1,509 participants included in the study are shown in Table 2. The mean MIBS-J total score was 1.85 (SD=2.37), the LA score was 0.85 (SD=1.37), and the AR score was 0.87 (SD=1.21). There were 748 (49.6%) primiparas with a mean EPDS total score of 4.67 (SD=3.89), ANX score of 2.22 (SD=1.98), ANH score of 0.19 (SD=0.58), and DEP score of 0.59 (SD=1.08). The mean number of supporters was 2.69 (SD=0.61), and 337 (22.6%) participants had problems with support. Univariate analysis showed that age, history of miscarriage, type of delivery, premature birth, problem with support, PPD, and EPDS total, ANX, ANH, DEP, MIBS-J total, LA, and AR scores were different between parities (p<0.01 for premature birth, ANH, and LA and p<0.001 for the other factors). There was no significant difference between the number of postpartum days and the MIBS-J total and EPDS total scores (p=0.492 and p=0.761, respectively).

**Table 2:** Participants’ demographic and background information. * p<0.05, ** p<0.01, *** p<0.001 SD: Standard deviation; CS: Cesarean section; MIBS-J: Mother-to-Infant Bonding Scale Japanese version; EPDS: Edinburgh Postnatal Depression Scale; PPD: Postpartum depression; NICU: Neonatal Intensive Care Unit; GCU: Growing Care Unit Number of days postpartum: when MIBS-J, EPDS, and social support questions were measured.

-	-	Total n=1509		Primipara n=748 (49.6%)		Multipara n=761 (50.4%)		
-	-	mean/n	(SD)/(%)	mean/n	(SD)/(%)	mean/n	(SD)/(%)	P-value
Age, mean (SD)		34.0	(4.83)	32.8	(5.20)	35.1	(4.14)	***
History of mental disorders, n (%)	Yes	136	(9.0)	78	(10.4)	58	(7.6)	-
	Never	1373	(91.0)	670	(89.6)	703	(92.4)	NS
History of miscarriage, n (%)	Yes	388	(25.7)	162	(21.7)	226	(29.7)	-
	Never	1121	(74.3)	586	(78.3)	535	(70.3)	***
Type of delivery, n (%)	Vaginal	900	(59.6)	433	(57.9)	467	(61.4)	-
	Instrumental	121	(8.0)	107	(14.3)	14	(1.8)	-
	Planned CS	303	(20.1)	83	(11.1)	220	(28.9)	-
	Emergency CS	185	(12.3)	125	(16.7)	60	(7.9)	***
Premature birth (<37 weeks), n (%)	Premature birth	100	(6.6)	35	(4.7)	65	(8.5)	-
	Full-term birth	1409	(93.4)	713	(95.3)	696	(91.5)	**
Number of supporters, mean (SD)		2.69	(0.61)	2.69	(0.61)	2.69	(0.61)	NS
Problems with support, n (%)	Yes	337	(22.3)	195	(26.1)	142	(18.7)	-
	No	1172	(77.7)	553	(73.9)	619	(81.3)	***
EPDS	Total, mean (SD)	4.67	(3.89)	5.47	(4.10)	3.89	(3.49)	***
	Anxiety, mean (SD)	2.22	(1.98)	2.71	(2.03)	1.73	(1.81)	***
	Anhedonia, mean (SD)	0.19	(0.57)	0.24	(0.65)	0.15	(0.48)	**
	Depression, mean (SD)	0.59	(1.08)	0.69	(1.19)	0.48	(0.96)	***
PPD (EPDS ≥9), n (%)	PPD	234	(15.5)	145	(19.4)	89	(11.7)	-
	Non-PPD	1275	(84.5)	603	(80.6)	672	(88.3)	***
MIBS-J	Total, mean (SD)	1.85	(2.37)	2.30	(2.56)	1.40	(2.08)	***
	Lack of affection, mean (SD)	0.85	(1.37)	0.94	(1.45)	0.76	(1.28)	**
	Anger and rejection, mean (SD)	0.87	(1.21)	1.20	(1.29)	0.55	(1.02)	***
Child's sex, n (%)	Female	723	(47.9)	347	(46.4)	376	(49.4)	-
	Male	786	(52.1)	401	(53.6)	385	(50.6)	NS
Admitted to NICU or GCU, n (%)	NICU or GCU	287	(19.0)	151	(20.2)	136	(17.9)	-
	Normal unit	1222	(81.0)	597	(79.8)	625	(82.1)	NS
Number of days postpartum, mean (SD)		32.6	(4.4)	32.5	(4.4)	32.6	(4.4)	NS

Multiple regression analysis 1

Table 3 shows the multiple regression analysis results of the relationship between MIB failure and parity, PPD, and social support. Parity, EPDS total score, number of supporters, and problems with support were significantly associated with the MIBS-J total and AR scores. Furthermore, EPDS total scores, number of supporters, and problems with support, but not parity, were associated with LA scores (p=0.832). The VIF was <1.7 for all independent variables, suggesting no multicollinearity issues.

**Table 3:** Results of multiple regression analyses between MIBS-J and EPDS, parity, and social support. * p<0.05, ** p<0.01, *** p<0.001 β: Standard partial regression coefficient; EPDS: Edinburgh Postnatal Depression Scale; MIBS-J: Mother-to-Infant Bonding Scale Japanese version; LA: Lack of Affection; AR: Anger and Rejection. All models are adjusted for age, history of mental disorders, history of miscarriage, type of delivery, premature birth, the child’s sex, and admission to the Neonatal Intensive Care Unit or Growing Care Unit.

-	MIBS-J total		MIBS-J LA		MIBS-J AR	
-	β	(95% CI)	β	(95% CI)	β	(95% CI)
Parity	0.086***	(0.038, 0.133)	0.006	(-0.047, 0,058)	0.172***	(0.125, 0.218)
EPDS total	0.453***	(0.405, 0.500)	0.305***	(0.252, 0.357)	0.447***	(0.399, 0.495)
Number of supporters	-0.116***	(-0.162, -0,070)	-0.082**	(-0.133, -0.031)	-0.096***	(-0.142, -0.050)
Problems with support	0.089***	(0.045, 0.134)	0.097***	(0.047, 0.146)	0.053*	(0.010, 0.097)

Effect modification

Table 4 shows the results of including the three interaction terms [parity×EPDS total], [parity×number of supporters], and [parity×problems with support] in the three multiple regression analysis models in “multiple regression analysis 1.” The interaction terms were included to examine whether the strength of the association between PPD symptoms and MIB and social support and MIB differs according to childbirth experience (Table 4). Only “parity×EPDS total” was associated with the dependent variable in each model (MIBS-J total score: β=0.080, p<0.001; LA: β=0.073, p<0.01; and AR: β=0.059, p<0.05; Table 4.

**Table 4:** Effect modification between parity and EPDS, number of supporters, and problems with support. * p<0.05, ** p<0.01, *** p<0.001 β: Standard partial regression coefficient; EPDS: Edinburgh Postnatal Depression Scale; MIBS-J: Mother-to-Infant Bonding Scale Japanese version; LA: Lack of Affection; AR: Anger and Rejection.

-	MIBS-J total		MIBS-J LA		MIBS-J AR	
-	β	(95% CI)	β	(95% CI)	β	(95% CI)
Parity×EPDS total	0.080***	(0.034, 0.126)	0.073**	(0.022, 0.124)	0.059*	(0.013, 0.104)
Parity×number of supporters	0.002	(-0.044, 0.047)	0.018	(-0.033, 0.068)	-0.022	(-0.067, 0.023)
Parity×problems with support	0.022	(-0.030, 0.075)	0.016	(-0.043, 0.075)	0.025	(-0.028, 0.077)

**Figure 1:**
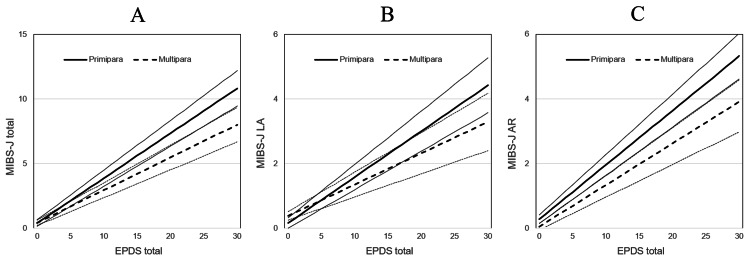
Single regression lines for EPDS total and MIBS-J total scores, with LA and AR scores, divided into groups of primiparas and multiparas. EPDS: Edinburgh Postnatal Depression Scale; MIBS-J: Mother-to-Infant Bonding Scale Japanese version; LA: Lack of Affection; AR: Anger and Rejection. Graph A: EPDS and MIBS-J total Primipara: y=0.406(0.152, 0.600)+0.347(0.310, 0.384)x, Multipara: y=0.420(0.219, 0.621)+0.253(0.215, 0.291)x Graph B: EPDS and LA Primipara: y=0.162(0.003, 0.322)+0.142(0.119, 0.165)x, Multipara: y=0.383(0.252, 0.514)+0.097(0.072, 0.122)x Graph C: EPDS and AR Primipara: y=0.279(0.148, 0.409)+0.168(0.149, 0.187)x, Multipara: y=0.049(-0.049, 0.147)+0.129(0.101, 0.148)x p<0.001 for the slopes of the two lines of all three graphs; values in parentheses are 95% CIs.

The participants were divided into primipara and multipara groups, and the simple linear regression lines were obtained (Figure 1). Multiple regression analysis was used to test the difference in slope, and the difference in the slopes of the two lines was significant, indicating that MIB failure was more severe in primiparas than in multiparas when PPD symptoms were more severe.

Multiple regression analysis 2

Table 5 shows the results of the relationship between the EPDS subscale scores and MIBS-J subscale scores. In both the crude and adjusted models, ANX, ANH, and DEP were significantly associated with the MIBS-J total, LA, and AR scores. However, the strength of the association in the adjusted model differed among the EPDS subscales. ANX scores (β=0.144, p<0.001) were somewhat strongly associated with LA scores, ANH scores (β=0.182, p<0.001) were strongly associated with LA scores, and ANX (β=0.221, p<0.001) and DEP (β=0.235, p<0.001) scores were strongly associated with AR scores.

**Table 5:** Results of multiple regression analyses between EPDS and MIBS-J at one month after birth. * p<0.05, ** p<0.01, *** p<0.001 β: Standard partial regression coefficient; EPDS: Edinburgh Postnatal Depression Scale; MIBS-J: Mother-to-Infant Bonding Scale Japanese version; LA: Lack of Affection; AR: Anger and Rejection. Adjusted models are adjusted for age, history of mental disorders, history of miscarriage, type of delivery, premature birth, the child’s sex, admission to the Neonatal Intensive Care Unit or Growing Care Unit, parity, number of supporters, and problems with support.

-		MIBS-J total		MIBS-J LA		MIBS-J AR	
		β	(95% CI)	β	(95% CI)	β	(95% CI)
EPDS anxiety	Crude	0.425***	(0.380, 0.471)	0.276***	(0.227, 0.324)	0.445***	(0.400, 0.491)
	Adjusted	0.206***	(0.153, 0.259)	0.144***	(0.085, 0.203)	0.221***	(0.169, 0.274)
EPDS anhedonia	Crude	0.362***	(0.315, 0.409)	0.294***	(0.246, 0.342)	0.292***	(0.244, 0.341)
	Adjusted	0.160***	(0.111, 0.209)	0.182***	(0.128, 0.236)	0.066**	(0.018, 0.115)
EPDS depression	Crude	0.420***	(0.374, 0.466)	0.257***	(0.208, 0.306)	0.435***	(0.389, 0.481)
	Adjusted	0.188***	(0.133, 0.243)	0.066*	(0.006, 0.126)	0.235***	(0.181, 0.289)

## Discussion

The present study revealed that being primipara was associated with elevated MIBS-J total and AR scores but not with LA scores, based on evaluations of MIB and PPD conducted around the first month postpartum. These findings are consistent with prior research, underscoring the idea that primipara status is a risk factor for MIB failure in Japan. Additionally, our results lend support to previous studies [7,9] that suggest parity enhances the emotions related to MIB in the first month postpartum. Although differences in the emotions evaluated across various studies complicate direct comparisons, a consistent observation is that primiparas are more likely to experience intensified negative emotions toward their child during the initial month postpartum.

Both the MIBS-J total and EPDS total scores were significantly higher for primiparas than for multiparas. Previous studies [14,15] have reported effect modification between parity, depression, and social support. To the best of our knowledge, the present study is the first to show an effect modification of the interaction between parity and PPD on MIB failure in the first postpartum month. Primiparas, new to the experience of childcare, often approach this role with limited familiarity, heightened sensitivity to stress, and a degree of naivety. Consequently, it becomes imperative to direct attention toward addressing MIB failure among primiparas, even if their experience of PPD is comparatively milder than that of multiparas. Conversely, the presence of effect modification within the interaction between parity and social support was not observed in this study, and social support seemed to improve MIB independent of parity. Several possible explanations underline the impact of parity on PPD and MIB [8,13]. The first explanation is related to endocrine and hormonal effects. Oxytocin and cortisol have been shown to affect MIB and cause PPD [30-32]. Primiparas have an enhanced cortisol response [33,34]. In two previous studies, multiparas experienced elevated oxytocin levels due to breastfeeding [35] and displayed elevated salivary oxytocin levels [36]; however, the sample sizes in both these studies were small at 10 and 36, respectively. In addition, mothers with a strong cortisol response exhibit aggressive (harsh) childcare behavior [37], which may be related to the primiparas' strong negative emotions. However, this study and all of the previous studies have not directly evaluated the relationship between parity and hormones and MIB or PPD. Therefore, future studies evaluating this relationship are warranted. The second explanation is related to the social and psychological impact of parity. A systematic review by McNamara J et al. [38] included 11 studies examining the relationship between parity and MIB, all conducted outside Japan, and reported that five studies identified primiparas as at risk for MIB failure. In comparison, the remaining six studies found no such relationship. Regarding PPD, studies from Portugal and Switzerland [39,40] reported that PPD symptoms and anxiety were milder in primiparas than in multiparas, studies from Brazil and the Czech Republic [41,42] reported that multipara was a risk factor for PPD, and a study from Canada [43] reported that parity was not related to PPD. We thought that parity had a psychological influence on mothers, and that this influence varied across countries, which may have led to these inconsistent results.

The third aspect pertains to mothers with high PPD levels or who experience MIB failure at their first birth, possibly being more unwilling to have a second child. The study by Tsuchida A et al. [9] and its supplemental data show that the mean EPDS total score for 32,342 primiparas was 5.62, and the mean MIBS-J total score was 1.93; however, the EPDS total and MIBS-J total scores at the first birth for 3,753 women with two births were 5.10 and 1.51, respectively. These scores were lower than those for primiparas, although statistical significance was not examined. These three causes could potentially coexist, align without contradiction, and may be interrelated. This study established significant associations among all subscales when examining the correlation between EPDS and MIBS-J subscales at the first month postpartum. However, significant differences were noted in the strength of the association between the subscales, with ANH scores being strongly and ANX scores being somewhat strongly associated with LA scores and ANX and DEP scores being strongly associated with AR scores. These findings complement previous results [7,17,18], stating that PPD symptoms affect the emotions involved in MIB differently. We remarkably found that ANX had a stronger association with MIB than did core symptoms of depression. Since the prevalence of ANX symptoms has been reported to be higher than that of PPD [44,45] and 10% of primiparas reported having ANX but not DEP [46], ANX is a symptom that should be noted regardless of DEP. Therefore, research on anxiety, which is more common and more strongly associated with MIB failure than depression, is desirable.

Limitations

The major limitation of this study is its retrospective nature, which restricts its capacity to establish causality. Therefore, a prospective study to examine the relationship between MIB, PPD, and parity over time would provide a better understanding. Since self-administered scales were adopted in this study, we could not know the actual prevalence of MIB failure and PPD. The two variables concerning social support are unique to this study and have not been validated. Furthermore, we were unable to gather data on factors influencing MIB, such as mothers' feelings about pregnancy [47], breastfeeding [48], and infant characteristics [49], as well as prenatal depressive symptoms [27] and fetal bonding [16]. The generalizability of our results is limited because this study population was derived from a single hospital.

## Conclusions

This study advances our understanding of the relationship between parity, PPD, and MIB failure in the first month postpartum. For the first time, it reveals the modifying effect of parity and PPD on MIB. Mothers, particularly primiparas, with high ANX and DEP scores (measured using the Japanese version of the EPDS) exhibited increased anger and rejection toward their children. Additionally, mothers with elevated ANH and ANX scores demonstrated less interest in their children. MIB failure was found to be more pronounced in primiparas than in multiparas, even when PPD was mild. This study holds value in identifying characteristics of mothers experiencing difficulties with MIB.
